# An unusual interventricular septal bounce in a patient with dermatomyositis: a case report

**DOI:** 10.1093/ehjcr/ytz034

**Published:** 2019-04-30

**Authors:** Vito Maurizio Parato, Davide Corradini, Andrea Di Matteo, Michele Scarano

**Affiliations:** 1 Cardiology Unit and EchoLab of Emergency Department, ‘Madonna del Soccorso’ Hospital, 3-7, Via Manara, San Benedetto del Tronto, Italy; 2 School of Medicine, ‘Politecnica delle Marche’ University, Ancona, Italy; 3 Clinica Reumatologica, “C. Urbani” Hospital, Università Politecnica delle Marche, Jesi, Ancona, Italy

**Keywords:** Dermatomyositis, Pericarditis, Pericardial constriction, Case report

## Abstract

**Background:**

In literature it has been reported in 1998, for the first time, a case of a 54-year-old man who developed constrictive pericarditis (CP) 12 years after diagnosis of dermatomyositis (DM). To our knowledge, this may be the only case reported.

**Case summary:**

A 16-year-old man presented to our institution because of symptoms posing a suspicion for an inflammatory disease; after physical examination, lab tests, and other investigations (electromyography, magnetic resonance, and muscular biopsy) was diagnosed as having DM. Patient also showed hepatomegaly and congested jugular veins: after clinical and imaging investigations (transthoracic echocardiography and transoesophageal echocardiography) he was diagnosed as having a CP. Patient underwent pericardial resection and the final outcome consisted of a completely regression of the symptoms.

**Discussion:**

Cardiac involvement in patients with DM ranges between 6% and 75%, and it can be clinically manifest or, far more frequently, sub-clinic. Pericardial involvement as a complication of DM is widely reported in the literature, but in almost all cases as acute pericarditis, effusive pericarditis or cardiac tamponade and almost never as a CP.

**Learning points**

- Constrictive pericarditis may be a complication of dermatomyositis.
- The interventricular septum bulging (or septal bounce) detected by echocardiography is the most striking finding of this type of complication.

## Introduction

Dermatomyositis (DM) is a systemic idiopathic inflammatory myopathy, characterized by an inflammatory infiltrate primarily affecting skeletal muscle and skin, with typical cutaneous lesions^2^ accompanying, or more often preceding, muscle weakness.

The reported incidence of DM ranges from 1.2 to 17 new cases per 1 000 000 inhabitants with a prevalence between 5 and 11 cases per 100 000 individuals.^3^ Dermatomyositis typically demonstrates a bimodal age distribution with both juvenile and adult forms.^4^

Pericardial involvement is less common in DM than other connective tissue diseases (<10%) and it nearly always manifests as acute pericarditis, pericardial effusion and cardiac tamponade.^5^ To our knowledge, only one published case of constrictive pericarditis (CP) in DM^6^ is available in the medical literature.

## Timeline

**Table:** 

15 January 2016	Hospitalization	
Clinical history	Constitutional symptoms (started 3 months before): progressive weakness and arthralgia	
Physical examination	Gottron’s papules Hepatomegaly Congested jugular veins BP 90/60 mmHg HR 82 b.p.m. (regular) Lower limb oedema	
Labs	Positive anti-Jo1 antibodies Increase of alanine transaminase and creatine phosphokinase	
Investigations (results)	Electromyography (pattern suggestive of dermatomyositis) Muscular magnetic resonance (generalized muscular oedema, atrophy, and adipose infiltration). Muscular biopsy (muscle fibre degeneration due to microvascular damage)	
Diagnosis	Dermatomyositis	
Treatment	Corticosteroids and methotrexate	
Time two (20 January 2016)	Cardiac evaluation	
Investigations and results	Electrocardiogram	Sinus rhythm and multiple ventricular premature beats
	Transthoracic echocardiogram	Interventricular septal bounce Significant reduction in mitral inflow velocity and an increase of tricuspid inflow velocity during inspiration
	Tissue Doppler imaging-E	Normal eʹ velocity from the medial mitral annulus (12 cm/s)
	Transoesophageal echocardiogram	Interventricular septal bounce Markedly thickened and calcified pericardium
Diagnosis	Constrictive pericarditis	
21 February 2016	Treatment	
Surgical treatment	Pericardial resection	
21 April 2016	Outcome	
	After pericardial resection cardiovascular symptoms disappeared and patient went back to normal life	
20 February 2018	2 years follow-up	
	Patient was asymptomatic with a NYHA class 1	

## Case presentation

A 16-year-old man was referred to our institution with progressive weakness and arthralgia, mainly involving proximal part of superior limbs. He developed these symptoms during the last 3 months in association with intermittent fever and weight loss. As the patient reported, a skin rash was present for 6 months. He had not received any therapy for these symptoms and signs.

At admission he had a temperature of 37.4°C. Physical examination demonstrated significant hepatomegaly. Mucocutaneous examination showed lichenoid papules on the dorsal surface of the hands, typical of Gottron papules.

His jugular venous pressure was raised and his heart sounds were normal. The chest was clear to auscultation. His blood pressure was 90/60 mmHg with a heart rate of 82 b.p.m. The respiratory rate was 16 breaths per minute and the oxygen saturation was 98%.

Blood investigations revealed:

- High alanine transaminase (78 IU/l) (reference range <35 IU/l)
- Very high creatine phosphokinase (736 UI/l) (reference range 60–174)
- Positive Rose-Waaler test.
- Positive anti-Jo1 antibody
- High NT-proBNP (3150 ng/l) (upper limit of normal: 900 ng/l).

In the proximal muscles, electromyography showed small, short, polyphasic actions potentials, with early recruitment motor unit action potentials, indicative of membrane irritability. These findings were more pronounced in the upper limbs. These findings suggested a diagnosis of DM.

A right quadriceps femoris biopsy showed B cells inflammatory infiltrated involving perivascular spaces and interfascicular septae, compatible with muscle fibre degeneration due to microvascular damage.

Muscular magnetic resonance showed generalized muscular oedema associated with atrophy and adipose infiltration.

The patient was diagnosed as having DM (Bohan and Peter’s criteria)^7^ and was started on corticosteroids therapy (1 mg/kg/day of oral prednisolone) together with methotrexate (15 mg, orally, once a week).

The 12-leads electrocardiogram showed a sinus rhythm with multiple ventricular premature beats.

The transthoracic echocardiogram (TTE) showed an atypical interventricular septal bounce into the left ventricular cavity because of ventricular interaction (Supplementary material online, *Video S1*). Left ventricle and right ventricle (RV) systolic function was normal.

Transoesophageal echocardiogram showed a markedly thickened and calcified pericardium and demonstrated very clearly the interventricular septal bounce (Supplementary material online, *Video S2*).

**Figure 1:**
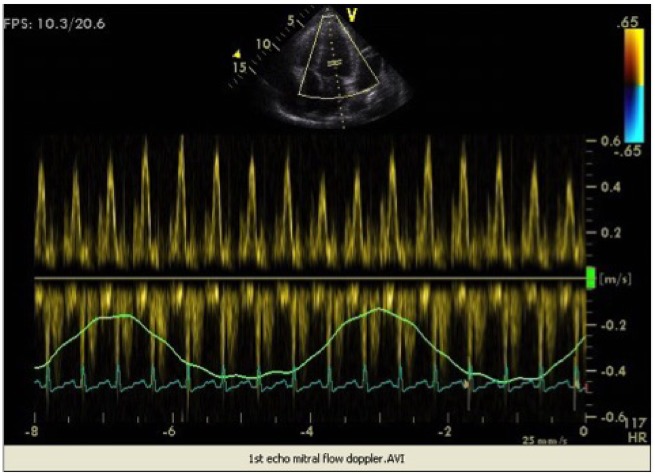
Respiratory variation of mitral inflow.

**Figure 2:**
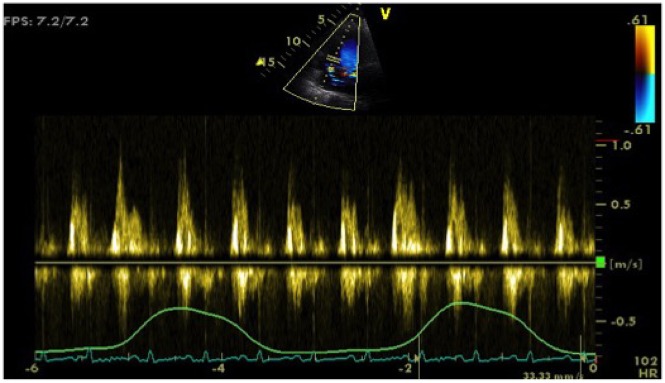
Respiratory variation of tricuspid inflow.

TTE-transvalvular PW Doppler flow studies showed a significant reduction of 31% in mitral inflow velocity and an increase of 25% in tricuspid inflow velocity during inspiration (*Figures 1*and*2*). The increase in tricuspid flow during inspiration was less significant than mitral inflow velocity reduction. In contrast, an increase of mitral inflow velocity was noted during expiration with a reduced filling velocity to the right heart (*Figures 1*and*2*). The aortic flow velocity decreased during inspiration and increased during expiration.

Tissue Doppler imaging (TDI) showed a medial eʹ velocity of 12 cm/s and a lateral eʹ velocity of 9 cm/s (annulus reversus). E/eʹ ratio (using average eʹ) was 9 (annulus paradoxus).

The echocardiographic findings were in keeping with constrictive pericarditis (CP).^8^

Patient was diagnosed as having a CP.

At the time of diagnosis the NYHA class was 3 (dyspnoea induced by mild exertion).

The patient underwent radical pericardiectomy by total median sternotomy without significant post-operative complications.

He was discharged on day 10 with a medical therapy consisting of diuretics (furosemide orally 20 mg BID); corticosteroids (5 mg per day of oral prednisone), methotrexate (15 mg orally, once per week), antibiotics (amoxicillin orally 3 g/day for 2 weeks) and paracetamol (maximum 4000 mg/day, orally). Hepatomegaly disappeared 4 weeks after operation together with alanine transaminase normalization.

After pericardial resection, the interventricular septum bulging and the respiratory variation of filling velocities completely disappeared (Supplementary material online, *Video S3*) (see also Table 1).

On follow-up 2 years later, the patient had no symptoms.

## Discussion

In 1899 Oppenheim reported for the first time, according to the current literature, cardiac involvement in polymyositis and DM.^9^

The pathophysiology of pericardial involvement is similar in all systemic inflammatory diseases. Immune complexes and inflammatory cytokines, such as vascular endothelial growth factor (VEGF) and basic fibroblast growth factor, seem to be related to pericardial involvement, even if their role is not completely cleared.^10^

Karatolios *et al*. report that the concentrations of VEGF in pericardial effusion and serum blood were significantly greater in subjects with systemic autoimmune inflammatory disease than in those with coronary artery disease. They also found VEGF levels in pericardial effusion positively correlated with markers of pericardial inflammation, such as lactate dehydrogenase and leucocytes.^11^ Chronic inflammation may lead to fibrosis, calcification and organization of pericardial fluid with a possible evolution towards pericardial constriction.^5^

Approximately 5–15% of patients with acute or recurrent pericarditis may have a systemic autoimmune disease, either overt or underlying. Pericardial involvement is common in all connective tissue diseases, rheumatoid arthritis and systemic vasculitis, but it might be less common in sarcoidosis and inflammatory bowel diseases. More specifically, while CP may complicate the rheumatoid arthritis, we know it is extremely rare in systemic lupus erythematosus.^12^

In DM, pericardial involvement usually expresses itself as an acute pericarditis, pericardial effusion, and cardiac tamponade.^5^

In 1998, Tamir *et al.*^6^ reported, for the first time, a case of a 54-year-old man who developed CP 12 years after the diagnosis of DM. The patient presented with progressive weakness, anasarca, and moderate anaemia. To our knowledge, this may be the only case like ours ever reported.

The patient’s clinical history may raise a discussion about the relatively rapid development of the constriction picture, within 3 months from the constitutional symptoms onset, even if the patient presented an indefinite skin rash for 6 months. The 2015 ESC Guidelines for the diagnosis and management of pericardial diseases^13^ reported that the delay between the initial pericardial inflammation and the onset of pericardial constriction is variable and that it is possible to define the clinical picture as persistent constriction after 3–6 months from the onset of symptoms.

Imazio *et al*.^14^ reported that a warning sign of a possible rapid evolution towards CP is a failure of empirical anti-inflammatory therapy. For this reason, in our patient, a lack of specific therapy in the previous 3 months may have lead to a rapid development of CP.

An important issue is how to correctly diagnose pericardial constriction.

Oh *et al*.^8^ described very accurately the echo-Doppler features of pericardial constriction compared with myocardial restriction.

In CP, the most striking findings are ventricular septal motion abnormalities (septal bounce), which do not occur in restriction.^15^ In the reported case, this finding allowed us to make an early diagnosis.

Respiratory variation in ventricular filling arises from the dissociation of intrathoracic and intracardiac pressure change and enhanced ventricular interaction in CP. As we know, inspiration reduces intrathoracic pressure which usually is fully transmitted to intracardiac pressures. On the contrary, in constriction, the intracardiac pressures fall much less than intrathoracic pressure because of pericardial constraint.

This difference in pressure change with inspiration results in reduced filling to left side of the heart. The reduction in left heart filling during inspiration causes a reduction in mitral inflow velocity and a shift of the interventricular septum towards the left ventricle. With expiration, left heart filling increases which shifts the interventricular septum back towards the RV, leading to reduced filling to right side of the heart and a late-diastolic reversal of flow in the hepatic veins.

In CP the eʹ velocity from TDI of the medial mitral annulus is 9 cm/s or greater while it is usually 6 cm/s or less in patients with a restrictive cardiomyopathy. Furthermore, the medial mitral annular eʹ velocity is usually greater than the lateral mitral annular eʹ, since the lateral motion of the heart is limited by the constrictive pericardium. This finding is recognized as “annulus reversus”.^8^ This again stands in contrast to what is expected in other forms of heart failure, and may reflect tethering of the lateral annulus by the constrictive process.^8^ Despite elevated LV filling pressures (restrictive transmitral flow pattern) E/e′ remains low in CP (annulus paradoxus). These findings, together with an inferior vena cava plethora and little respiratory variations, are important echocardiographic signs of pericardial constriction.^8^ As reported in the European Association of Cardio-Vascular Imaging (EACVI) position paper,^15^ other imaging techniques are useful to make a diagnosis of pericardial constriction. Cardiac computed tomography and cardiac magnetic resonance (CMR) usually detect very accurately pericardial calcification and, in a clinical setting of suspected constriction, they have an essential role in making a correct diagnosis. However, calcification alone does not allow making a diagnosis of ‘pericardial constriction’. In addition, real-time cine CMR demonstrates dilated right atrium, elongated RV and ventricular interdependence.^15^ At the same time, pericardial adhesions between the thickened pericardium and the epicardial surface of the myocardium with reduced mobility of the myocardium may be highlighted by tagged cine CMR imaging.^15^

## Conclusion

It is well known that, among cardiovascular complications due to DM, pericardial involvement is rare and usually it manifests as acute pericarditis, pericardial effusion, and/or cardiac tamponade. The reported case demonstrates that CP is a possible complication of DM. A very important point is to correctly using all imaging techniques today available in order to make a correct diagnosis of pericardial constriction.

## Supplementary Material

ytz034_Supplementary_Video
