# Supplementary material for: An unusual interventricular septal bounce in a patient with dermatomyositis: a case report
Source: Eur Heart J Case Rep. 2019 Apr 30;3(2):ytz034. doi: 10.1093/ehjcr/ytz034 (PMC6601201; doi:10.1093/ehjcr/ytz034)
Supplement: ytz034_Supplementary_Video [file ytz034_supplementary_video.zip › ytz034-suppl_data/ytz034_Slide_Set.pptx]

## Slide 1
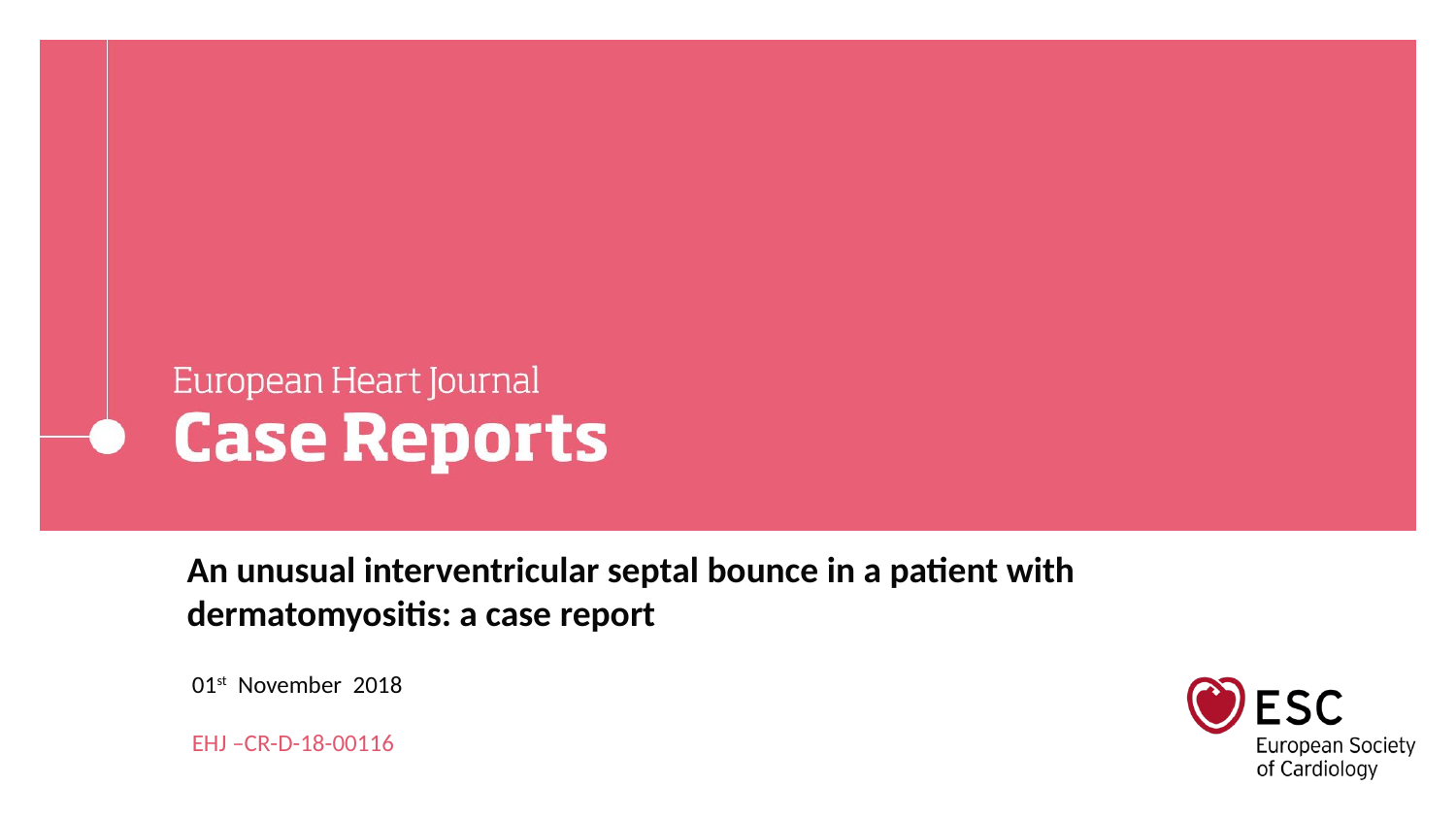

# An unusual interventricular septal bounce in a patient with dermatomyositis: a case report
01st November 2018
EHJ –CR-D-18-00116

## Slide 2
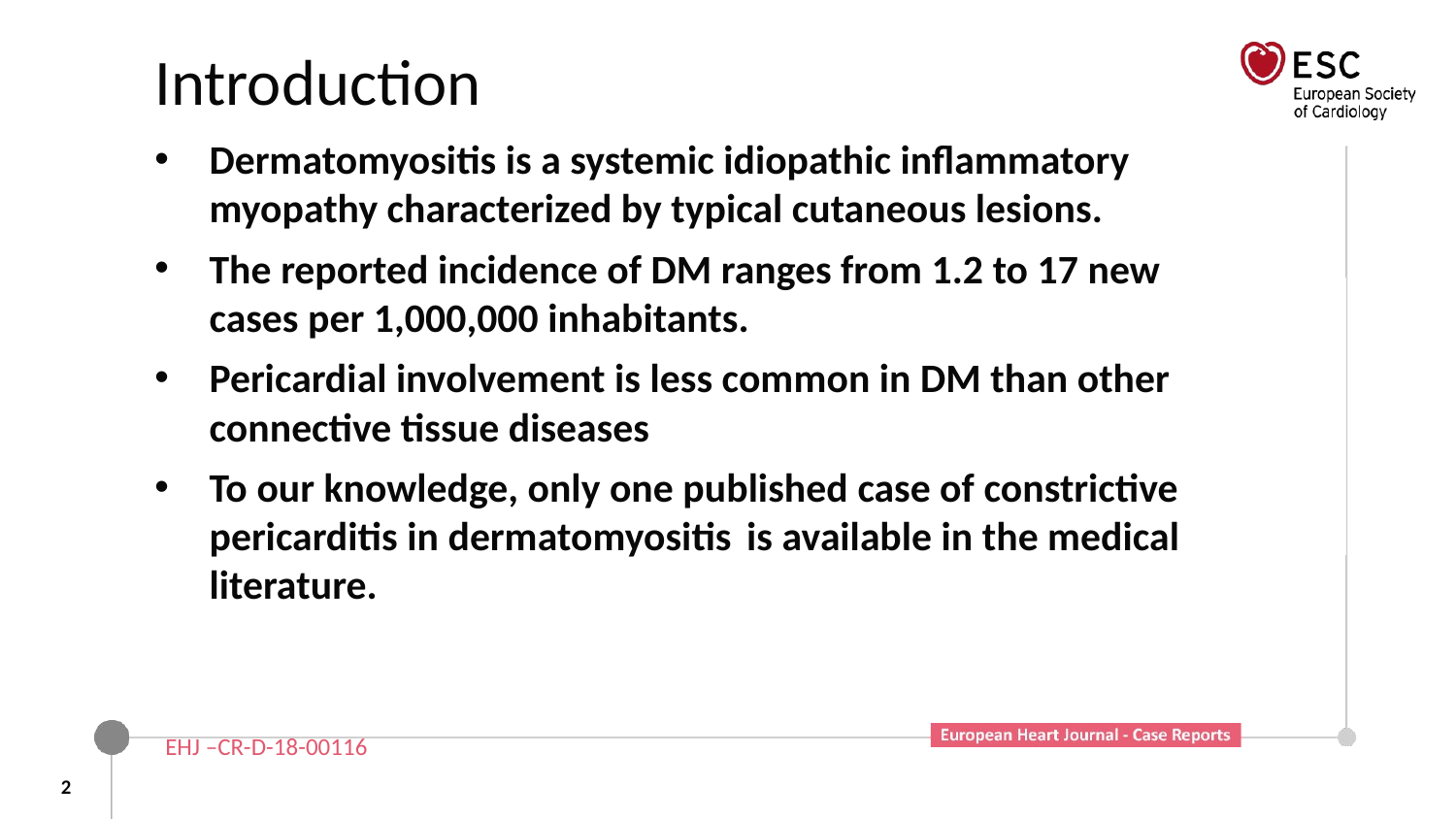

# Introduction
Dermatomyositis is a systemic idiopathic inflammatory myopathy characterized by typical cutaneous lesions.
The reported incidence of DM ranges from 1.2 to 17 new cases per 1,000,000 inhabitants.
Pericardial involvement is less common in DM than other connective tissue diseases
To our knowledge, only one published case of constrictive pericarditis in dermatomyositis is available in the medical literature.
EHJ –CR-D-18-00116
2

## Slide 3
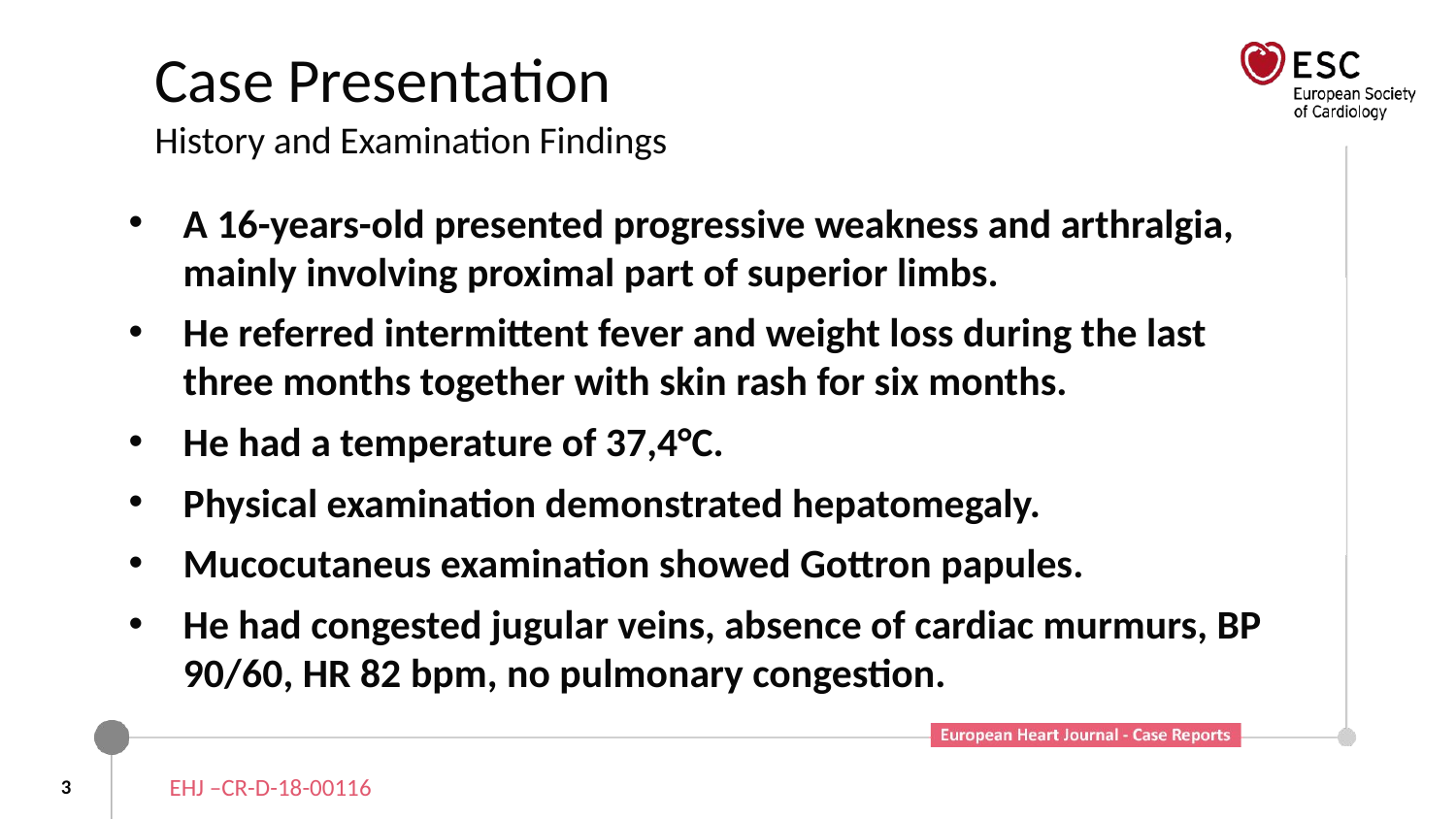

# Case Presentation History and Examination Findings
A 16-years-old presented progressive weakness and arthralgia, mainly involving proximal part of superior limbs.
He referred intermittent fever and weight loss during the last three months together with skin rash for six months.
He had a temperature of 37,4°C.
Physical examination demonstrated hepatomegaly.
Mucocutaneus examination showed Gottron papules.
He had congested jugular veins, absence of cardiac murmurs, BP 90/60, HR 82 bpm, no pulmonary congestion.
3
EHJ –CR-D-18-00116

## Slide 4
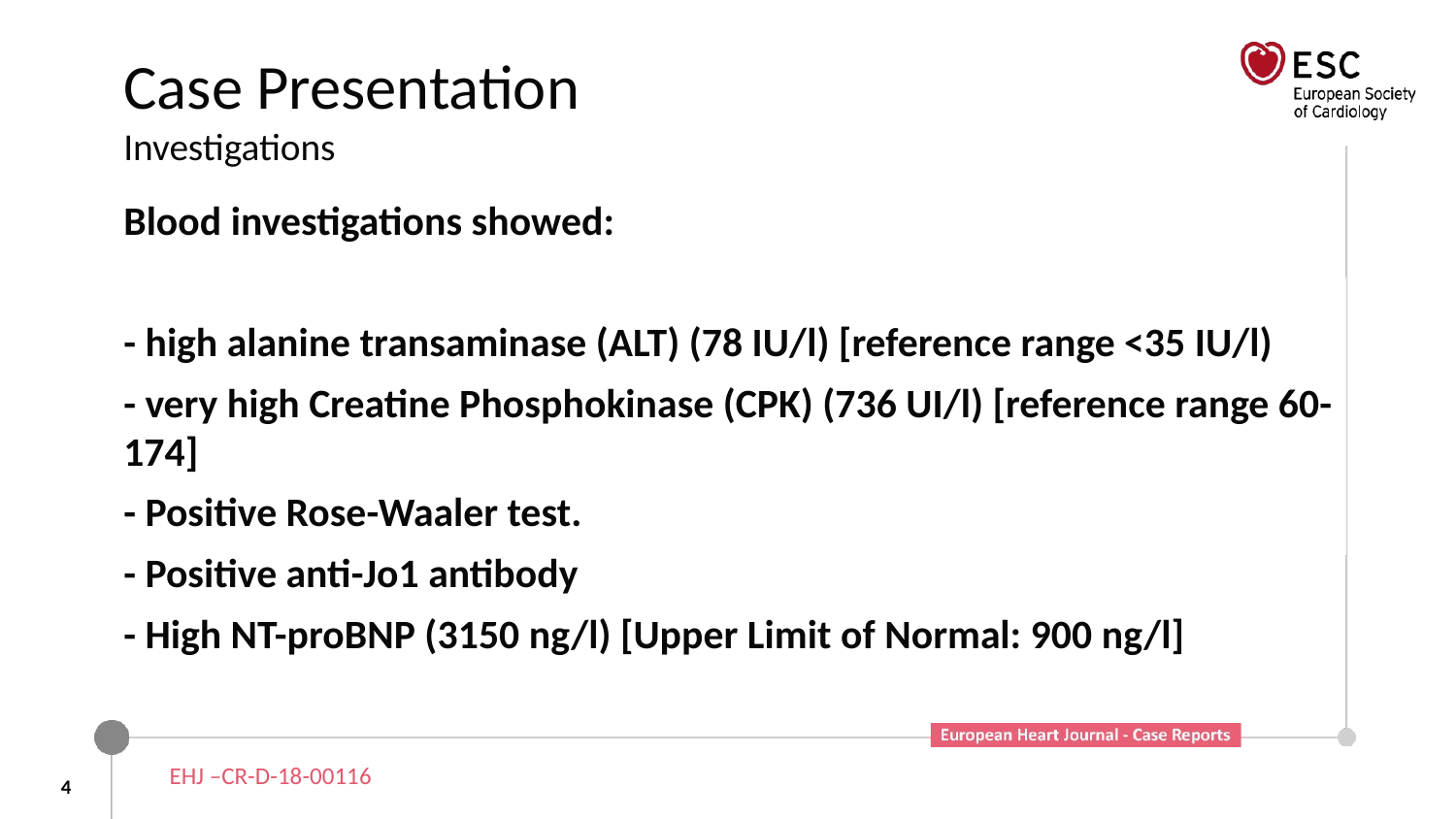

# Case Presentation Investigations
Blood investigations showed:
- high alanine transaminase (ALT) (78 IU/l) [reference range <35 IU/l)
- very high Creatine Phosphokinase (CPK) (736 UI/l) [reference range 60-174]
- Positive Rose-Waaler test.
- Positive anti-Jo1 antibody
- High NT-proBNP (3150 ng/l) [Upper Limit of Normal: 900 ng/l]
4
EHJ –CR-D-18-00116

## Slide 5
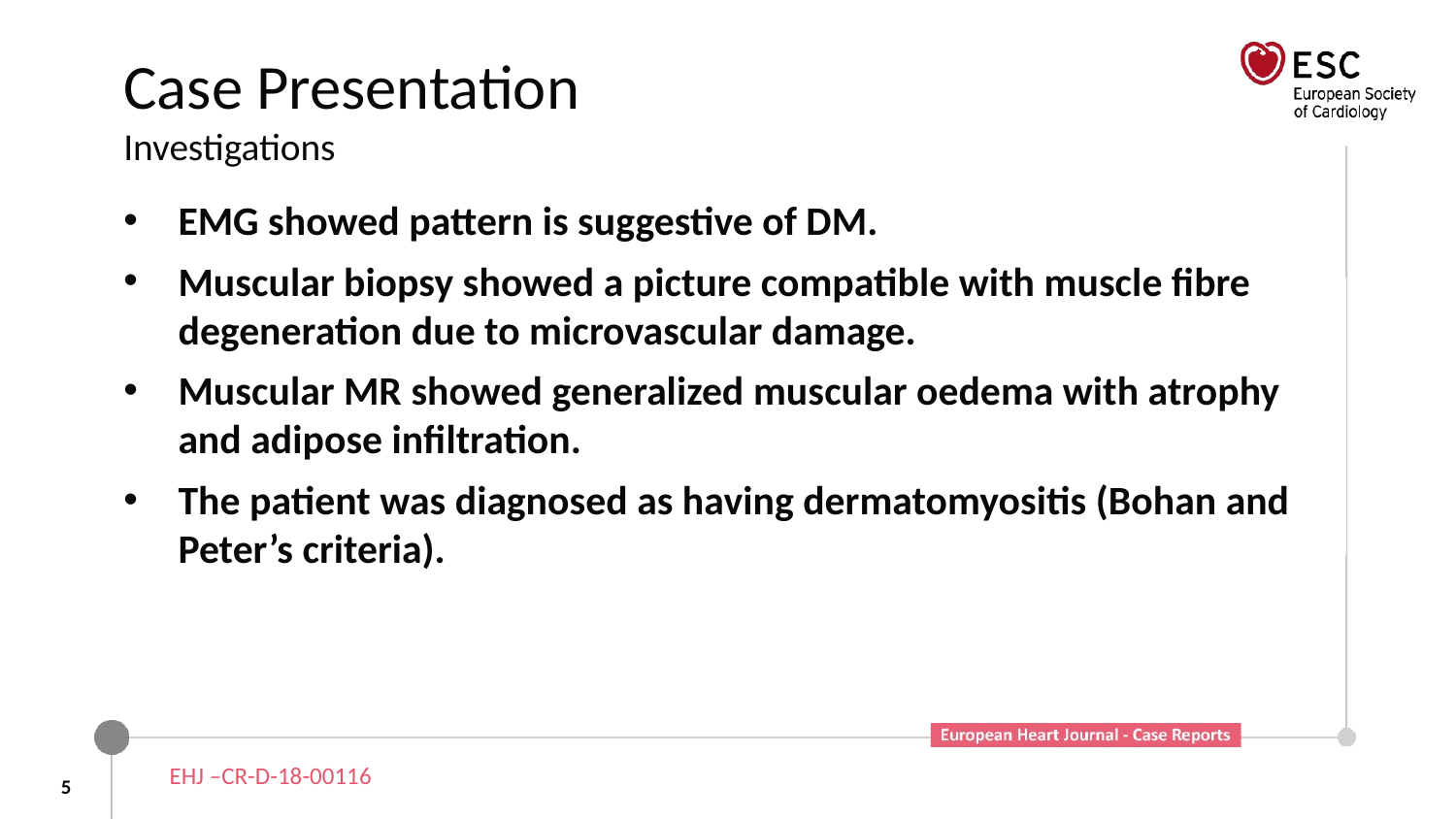

# Case Presentation Investigations
EMG showed pattern is suggestive of DM.
Muscular biopsy showed a picture compatible with muscle fibre degeneration due to microvascular damage.
Muscular MR showed generalized muscular oedema with atrophy and adipose infiltration.
The patient was diagnosed as having dermatomyositis (Bohan and Peter’s criteria).
5
EHJ –CR-D-18-00116

## Slide 6
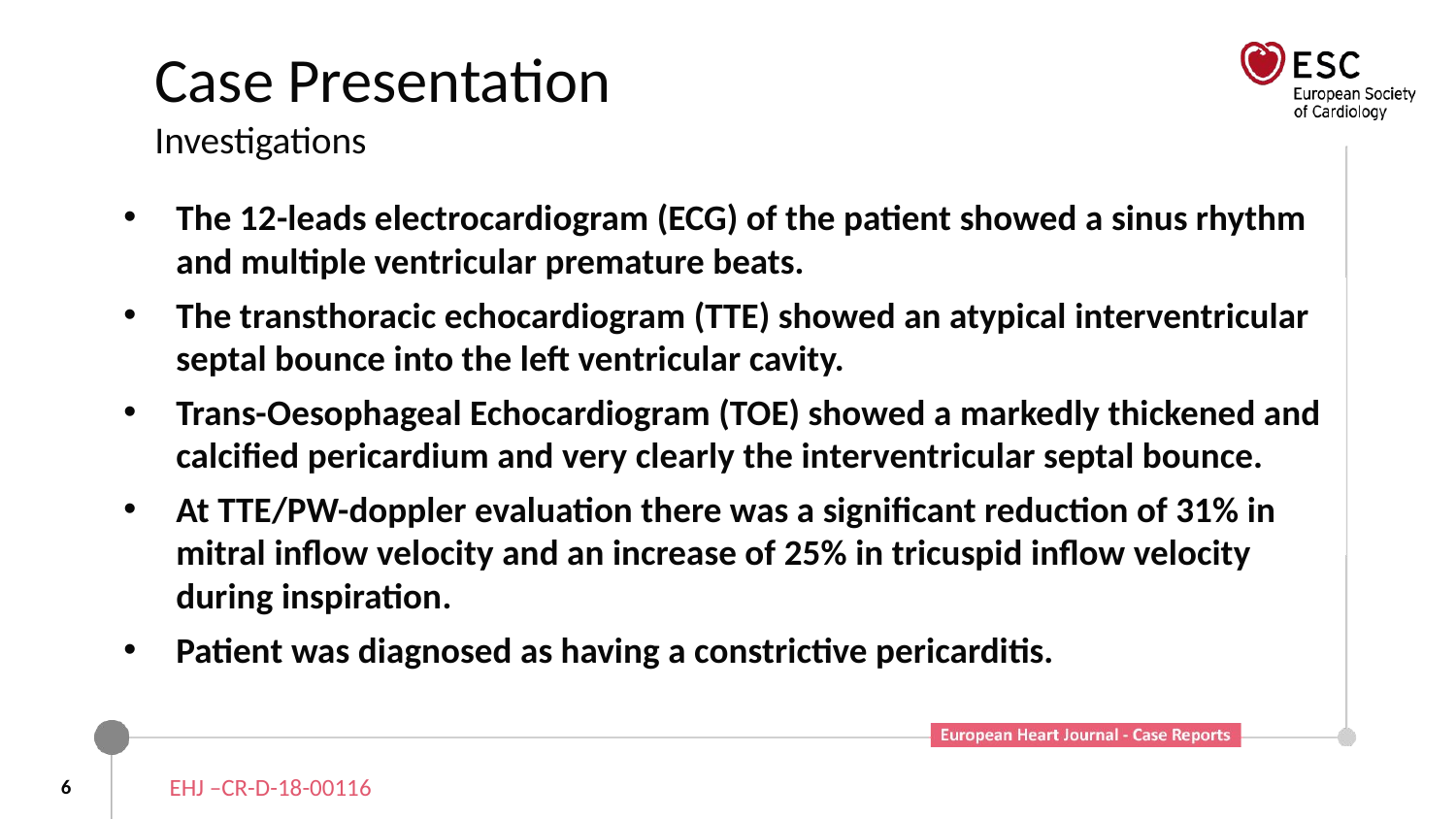

# Case Presentation Investigations
The 12-leads electrocardiogram (ECG) of the patient showed a sinus rhythm and multiple ventricular premature beats.
The transthoracic echocardiogram (TTE) showed an atypical interventricular septal bounce into the left ventricular cavity.
Trans-Oesophageal Echocardiogram (TOE) showed a markedly thickened and calcified pericardium and very clearly the interventricular septal bounce.
At TTE/PW-doppler evaluation there was a significant reduction of 31% in mitral inflow velocity and an increase of 25% in tricuspid inflow velocity during inspiration.
Patient was diagnosed as having a constrictive pericarditis.
6
EHJ –CR-D-18-00116

## Slide 7
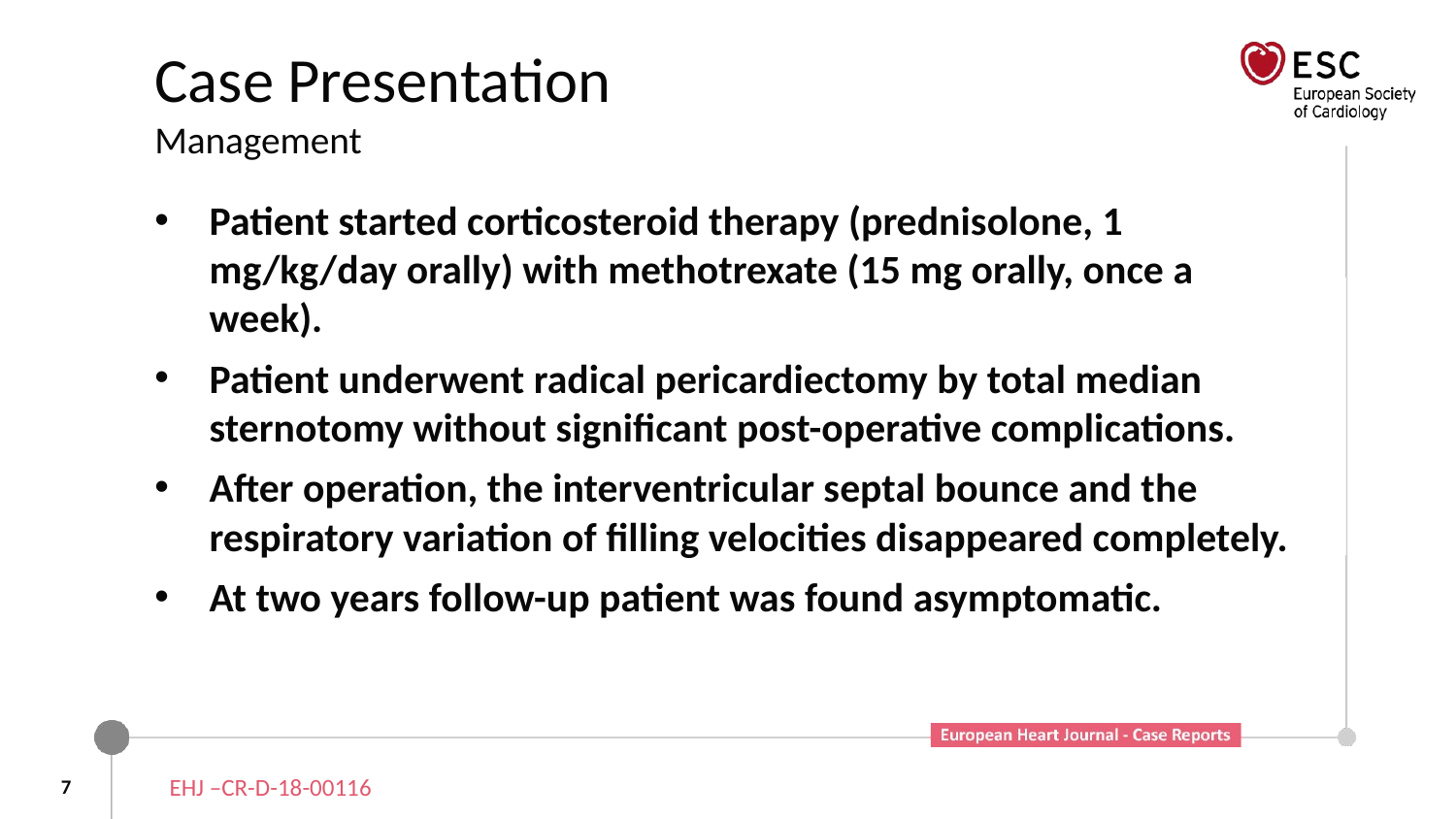

# Case Presentation Management
Patient started corticosteroid therapy (prednisolone, 1 mg/kg/day orally) with methotrexate (15 mg orally, once a week).
Patient underwent radical pericardiectomy by total median sternotomy without significant post-operative complications.
After operation, the interventricular septal bounce and the respiratory variation of filling velocities disappeared completely.
At two years follow-up patient was found asymptomatic.
7
EHJ –CR-D-18-00116

## Slide 8
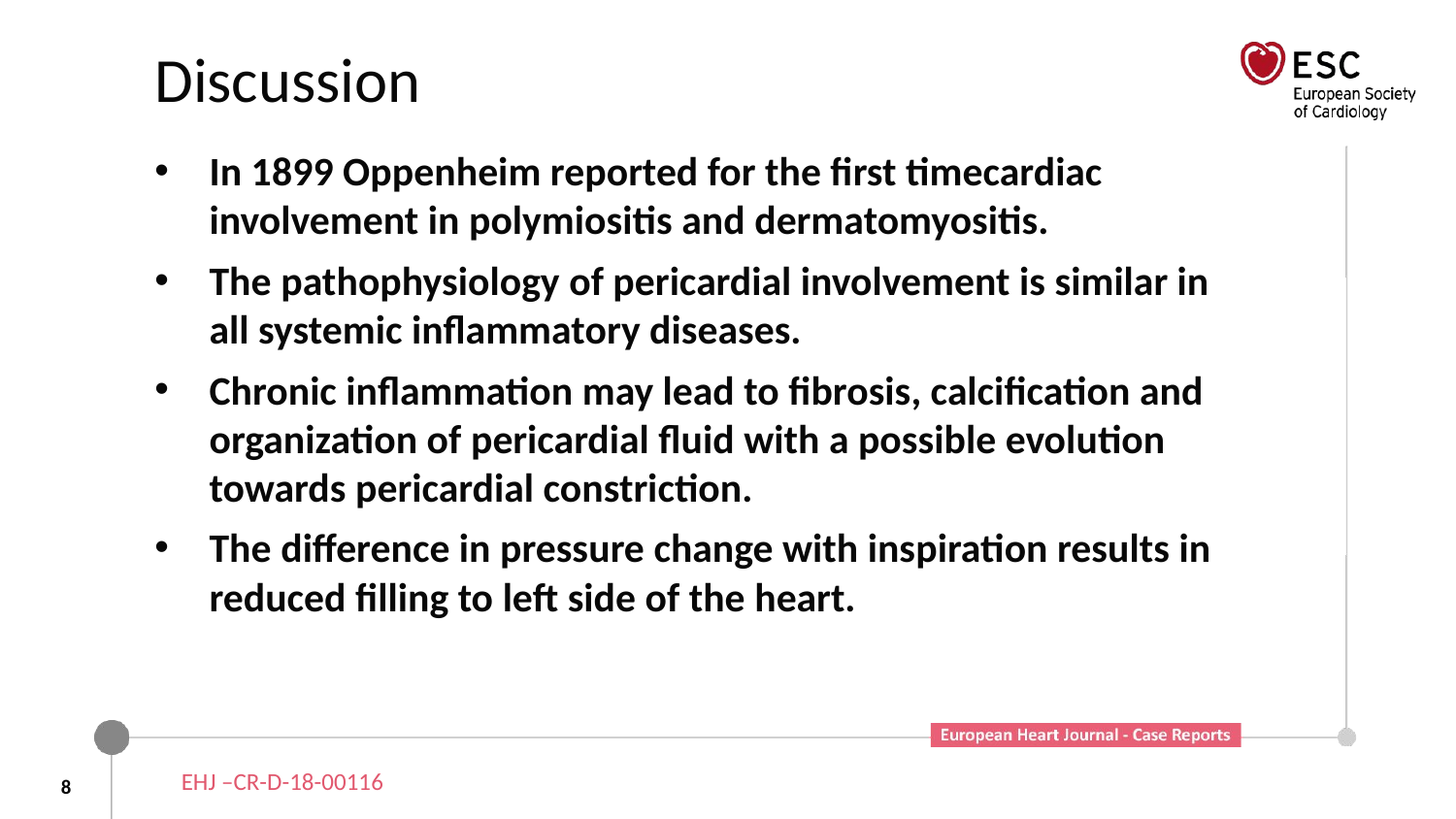

# Discussion
In 1899 Oppenheim reported for the first timecardiac involvement in polymiositis and dermatomyositis.
The pathophysiology of pericardial involvement is similar in all systemic inflammatory diseases.
Chronic inflammation may lead to fibrosis, calcification and organization of pericardial fluid with a possible evolution towards pericardial constriction.
The difference in pressure change with inspiration results in reduced filling to left side of the heart.
EHJ –CR-D-18-00116
8

## Slide 9
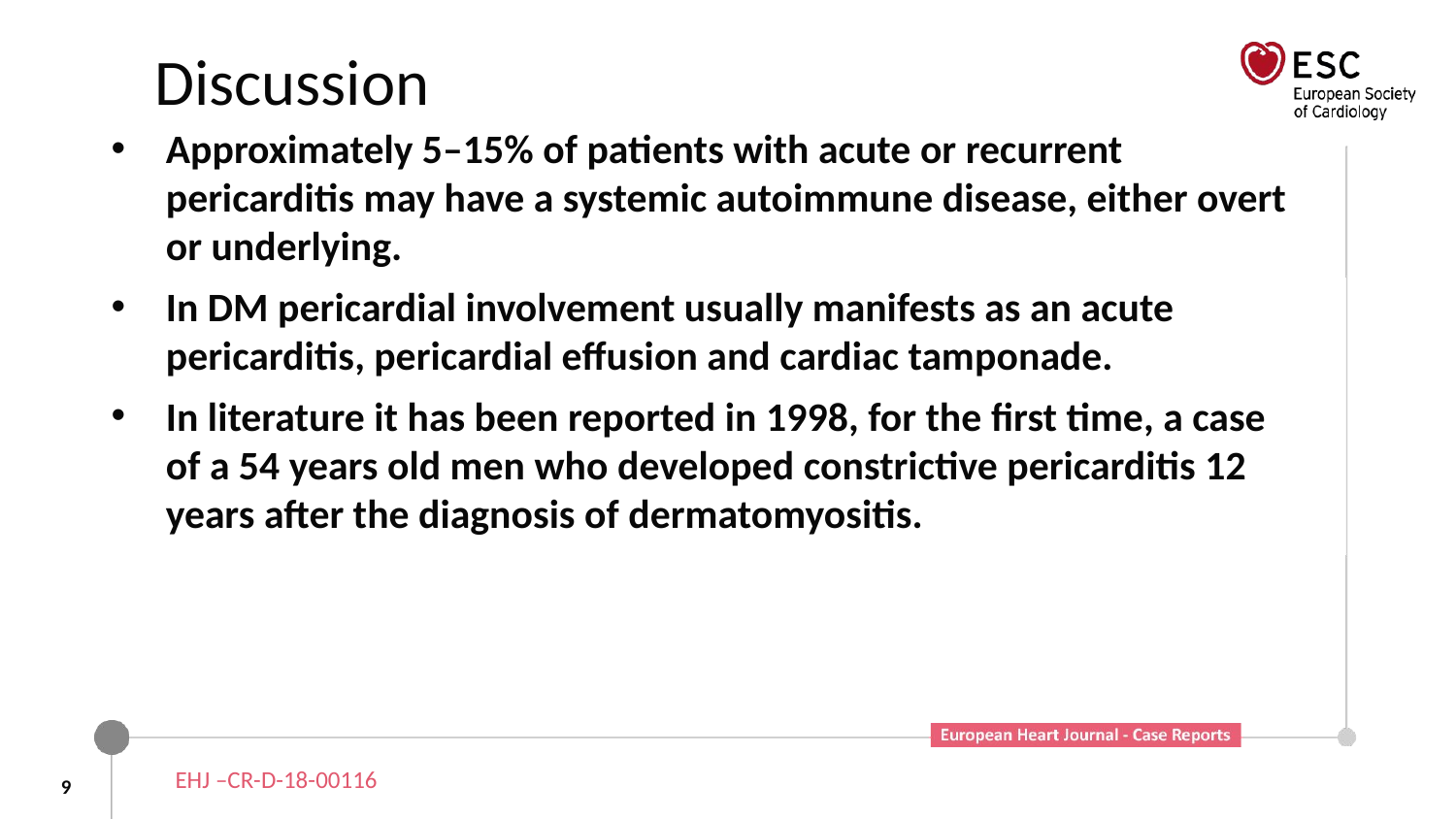

# Discussion
Approximately 5–15% of patients with acute or recurrent pericarditis may have a systemic autoimmune disease, either overt or underlying.
In DM pericardial involvement usually manifests as an acute pericarditis, pericardial effusion and cardiac tamponade.
In literature it has been reported in 1998, for the first time, a case of a 54 years old men who developed constrictive pericarditis 12 years after the diagnosis of dermatomyositis.
EHJ –CR-D-18-00116
9

## Slide 10
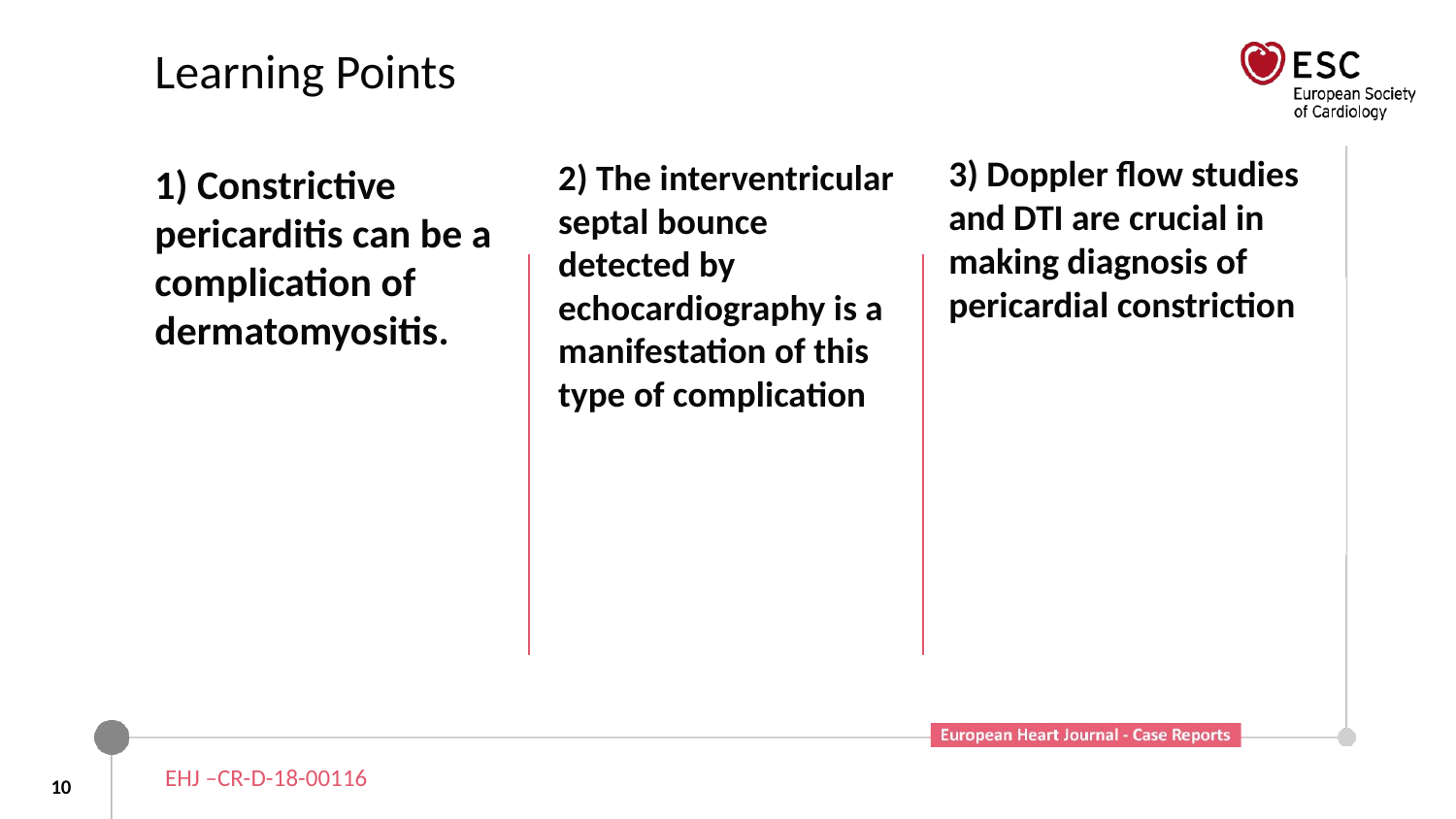

# Learning Points
3) Doppler flow studies and DTI are crucial in making diagnosis of pericardial constriction
2) The interventricular septal bounce detected by echocardiography is a manifestation of this type of complication
1) Constrictive pericarditis can be a complication of dermatomyositis.
EHJ –CR-D-18-00116
10

## Slide 11
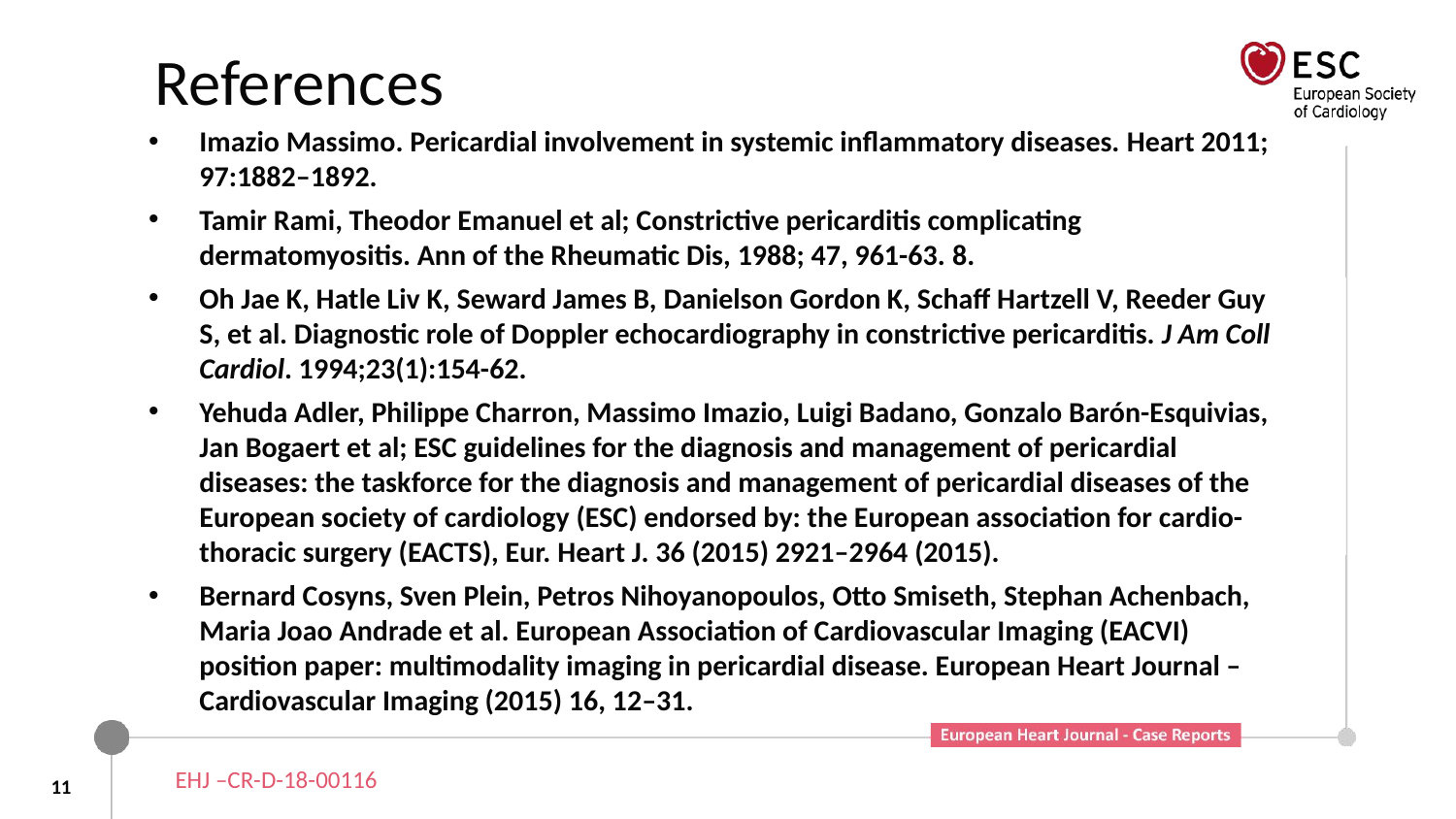

# References
Imazio Massimo. Pericardial involvement in systemic inflammatory diseases. Heart 2011; 97:1882–1892.
Tamir Rami, Theodor Emanuel et al; Constrictive pericarditis complicating dermatomyositis. Ann of the Rheumatic Dis, 1988; 47, 961-63. 8.
Oh Jae K, Hatle Liv K, Seward James B, Danielson Gordon K, Schaff Hartzell V, Reeder Guy S, et al. Diagnostic role of Doppler echocardiography in constrictive pericarditis. J Am Coll Cardiol. 1994;23(1):154-62.
Yehuda Adler, Philippe Charron, Massimo Imazio, Luigi Badano, Gonzalo Barón-Esquivias, Jan Bogaert et al; ESC guidelines for the diagnosis and management of pericardial diseases: the taskforce for the diagnosis and management of pericardial diseases of the European society of cardiology (ESC) endorsed by: the European association for cardio-thoracic surgery (EACTS), Eur. Heart J. 36 (2015) 2921–2964 (2015).
Bernard Cosyns, Sven Plein, Petros Nihoyanopoulos, Otto Smiseth, Stephan Achenbach, Maria Joao Andrade et al. European Association of Cardiovascular Imaging (EACVI) position paper: multimodality imaging in pericardial disease. European Heart Journal – Cardiovascular Imaging (2015) 16, 12–31.
EHJ –CR-D-18-00116
11
